# Cervical Cancer: Early Detection and Prevention in Reproductive Age Group

**DOI:** 10.7759/cureus.31312

**Published:** 2022-11-09

**Authors:** Sakshi Basoya, Ashish Anjankar

**Affiliations:** 1 Medicine, Jawaharlal Nehru Medical College, Datta Meghe Institute of Medical Sciences, Wardha, IND; 2 Biochemistry, Jawaharlal Nehru Medical College, Datta Meghe Institute of Medical Sciences, Wardha, IND

**Keywords:** cervical cancer, vaccine, lifestyle modifications, reproductive age group, pap smear

## Abstract

Cancer has been one of the major illnesses faced by people over many generations. Despite the advancements made in medicine, there are still many problems faced by humankind. Every year a large number of people are diagnosed with cervical cancer. It is the most common type of cancerous condition prevalent among females, especially females over thirty years of age. Like any other cancer, cervical cancer also occurs because of the rapid uncontrolled division of the body cells. A primary reason for its development is the longstanding infection of certain types of *human papillomavirus* (HPV). A screening test is done to confirm the presence of the virus in the cervix. Many screening tests are available today for accurate diagnosis or confirmation of the condition being suffered from. The main goal of screening is early detection and making certain lifestyle changes to deduce the potential harm of the disease and start the treatment as soon as possible. A thorough study of the already published articles by scholars, professors, and doctors is carried out to conclude the necessity of cervical cancer screening and early detection.

## Introduction and background

The fourth most common cancer in women worldwide, cervical cancer kills 85% of its victims in low- and middle-income countries [1]. In developing countries, cervical cancer can account for up to 25% of all cancers in women, making it typically the most common disease affecting females. 2012 saw the discovery of 528,000 new instances of cervical cancer, 123,000 of which were in India. Approximately 266,000 women died from this illness in 2012, 67,000 of whom died in India [1,2]. The percentage of new cases and deaths that occur in low-resource areas or among members of socially and economically disadvantaged sectors of the population is about 85% and 90%, respectively [2]. Unprotected intercourse, polygamy, low socioeconomic status, early marriages, inadequate education, early menstrual cycles, many pregnancies, smoking, co-infections, HPV infections, changed hormones, and a weakened immune system have all been recognized as risk factors for cervical cancer. High-risk human papillomavirus (HPV) types 16, 18, and 31 are sexually transmitted infections (STIs) that are carcinogenic and cause cervical cancer [3]. It is discovered that carcinogenic human papillomavirus (HPV) infection is linked to the majority of cervical cancer incidences [1,4]. Since the prevalence of HPV infection appears to be steadily rising, this issue is still crucial [4]. Although cervical cancer is one of the most prevalent diseases affecting women in low- and middle-income countries, both primary and secondary preventive strategies, including immunization against the human papillomavirus and preinvasive disease therapy, are viable. The Ministry of Health has called for a coordinated strategy to end cervical cancer, which entails a difficult set of actions at every level of a health service. This article describes the situation of cervical cancer prevention in low- and middle-income countries, the innovations being used to improve results, and a discussion of the next steps required as we progress towards worldwide eradication.

## Review

Human papillomavirus

Papillomaviruses are pervasive, have been found in a wide range of species in addition to humans, and are unique to the hosts they infect. If treatment is delayed, HPV infection can occasionally develop into a premalignant lesion that becomes more advanced and aggressive [3]. Numerous variables were found to be associated with the awareness of cervical cancer [5]. More significant than the correlation between smoking and lung cancer is the strength of the connection between HPV and cervical squamous cell carcinoma [6]. Based on genetic variations revealed by DNA sequence data, more than 200 different varieties of HPV have been detected. HPV is linked to various clinical conditions, from benign lesions to cancer [4]. Consistent infections can cause lesions occurring before cancer and invasive cancer in a small percentage of women, even though most conditions go away without causing harm [7]. Asymptomatic lesions seen during routine cervical cancer screenings range in size from those to significant lesions on the vulva, vagina, cervix, and specific extra genital locations. Varies with age, location, and gestational age, its frequency in pregnancy ranges from 5.5% to 65% (increasing with gestational age) [8]. Pregnancy-related infections have been linked to unfavorable effects, including spontaneous miscarriage, premature delivery, placental abnormalities, and fetal development limitation. But there is a variety of data to support these negative impacts [8-10]. Promiscuous sexual behavior, reproductive variables such as genital cleanliness, early menarche, the window between menarche and the first sexual encounter, young marriage age, high parity, other sexually transmitted infections, and smoking are among the other risk factors for cervical cancer. After starting sexual activity in their 20s, women experience the highest rate of HPV infection. Typically, early invasive cervical cancer develops after a chronic HPV infection for 10 years [1]. Human immunodeficiency virus (HIV) infection increases the likelihood of persistent human papillomavirus (HPV) infection, which raises the incidence of intraepithelial lesions and raises the risk of invasive cervical cancer in HIV-positive individuals [11].

Early detection and screening

Early identification of cervical cancer (CC) has decreased the disease's mortality and morbidity, and both structured and opportunistic pap smear-taking has been shown to minimize CC incidence rates [10]. Effective screening programs can help prevent cervical cancer. Attempts to prevent HPV infection, such as condom usage and sexual education for young people, as well as the HPV vaccine, are the primary methods of preventing this type of neoplasm [12]. Cervical cell screening and DNA-HPV testing in high-risk populations are used as a secondary method of preventing cervical cancer by identifying precancerous lesions or tumors in their early stages. Some international organizations recommend co-testing, which involves using these two tests. The most excellent prevention, meanwhile, is immunization rather than screening in teens and young adults [12]. One of the most effective public health preventative programs has been cervical screening. According to reports, various variables affect screening results, including facility accessibility, the accuracy of screening tests, the effectiveness of follow-up care, and the care and management of lesions found [13]. Cytology served as the chief screening method for 50 years, and cervical intraepithelial lesions (CIN) were surgically removed when they were found to be cancerous [14]. The utilization of preventative and diagnostic information is significantly influenced by cancer health learning, which would be the capacity to see, comprehend, and use health knowledge to form wise health decisions [15]. Due to a shortage of qualified healthcare professionals and inadequate funding for screening programs, low- and middle-income nations have deficient levels of cervical cancer screening services [13]. Identifying the HPV infection (HPV) as the principal reason for cervical cancer fundamentally altered theories on how to combat the illness. Around 1990, testing with HPV began, and prophylactic HPV vaccination received its license in 2006 [14]. Health professionals ought to focus more on populations that are more likely to have poor health literacy. Table 1 shows high- and low-risk HPV risk in relation to different age groups.

**Table 1:** High and low- risk HPV risk in relation to different age groups. x^2^ test used, p=0.345 This table has been taken from source [16]

Age groups	Risk groups				
	Unknown	High- risk	Low- risk	Mixed	Total
11-20	0(0.0%)	0(0.0%)	3(3.3%)	1(1.1%)	4(4.4%)
21-30	0(0.0%)	2(2.2%)	18(20.0%)	2(2.2%)	22(24.4%)
31-40	0(0.0%)	6(6.7%)	18(20.0%)	1(1.1%)	25(27.8%)
41-50	0(0.0%)	6(6.7%)	13(24.4%)	2(2.2%)	21(23.3%)

Two sections that indicated a tendency toward less comprehensive screening were highlighted when key guidelines-making organization unified their recommendations for cervical cancer screening in 2012: 1) Regardless of the age at which sexual activity first begins, regular cervical screening really shouldn't start until age 21, and 2) The recommended frequency for regular cytology testing would be every three years, with the option to transition to cytology plus HPV testing ("co-testing") every five years starting at age 30. These modifications were brought about by a greater comprehension of how the sexually transmitted infection of human papillomavirus (HPV), which is quite common, contributes to cervical cancer development [17]. Associated with cancer screening programs and HPV vaccination campaigns supported by substantial public spending, human papillomavirus incidence rates and mortality rates have steadily decreased in industrialized nations [18]. By conducting routine Pap-smear tests, cervical cancer incidence can be decreased. A professional cytopathologist must manually analyze hundreds of sub-images on a single slide under a microscope for each patient throughout screening, which takes time, is arduous, and is prone to mistakes. The gold standard diagnostic test for asymptomatic females is the Pap (Papanicolaou) smear, which in solid healthcare systems may reduce the average annual mortality rate from cervical cancer by 2.6 percent. One of the most reliable screening methods for spotting cervical cancer at an early stage is the Pap smear [3]. Pap smear sensitivity is 70% for squamous intraepithelial lesions or aggressive tumor detection. Together with HPV, DNA testing offers high sensitivity for the early detection of premalignant lesions. It is common knowledge that prevention is preferable to treatment. According to several types of research undertaken globally, the burden of cervical cancer is being reduced via awareness and education campaigns. In this regard, the influence of knowledge on healthy behavior and lifestyle adjustment has been researched to prevent cervical cancer and human papillomavirus contamination [3].

Pap/CISOE-A, a modified Papanicolaou classification system, or the Bethesda classification system, is used to classify cervical smears [19]. It has been debated if the drawbacks outweigh the minimal decrease in cervical cancer fatalities, even though the implementation of nationwide screening programs has reduced cervical cancer rates. Both techniques have comparable sensitivity to the Pap test and somewhat higher specificity for diagnosing high-grade cervical neoplasm (CIN II/III or HSIL and cervical cancer) in an initial screening scenario [19]. Easy-to-use screening methods include the direct inspection with an eye of the cervix with 5 percent acetic acid (VIA) and the Pap smear. The women who test positive might then be sent to the same hospital for a colposcopy evaluation and further care. To deal with the problem of loss to follow-up (LTFU), which is frequently experienced in these settings, it is simple to use the WHO-recommended "Screen and Treat" strategy [7]. The ideal situation would be for local healthcare standards of practice and regional health systems policy to address the much more common implementation challenges in a particular environment to maximize effect and ensure program sustainability. Acceptability, acceptance, appropriateness, and feasibility are vital in a community health system for facilitating the implementation of long-lasting health services of the highest caliber, easily accessible and oriented to the needs of the individual.

The Pap smear method

**Figure 1:**
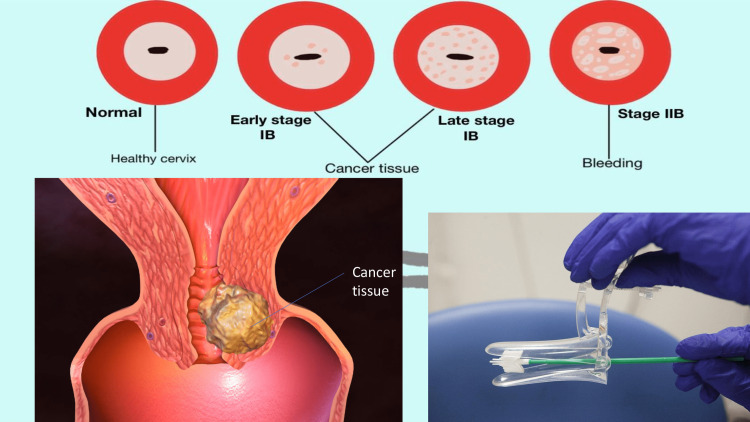
Cervical cancer screening method This figure is the sole creation of the author.

The classic Pap-smear is made in a straightforward and uncomplicated manner; biological material first from the squamocolumnar connection in the cervix is gently scraped off with a brush or scraper and spread onto a microscope slide measuring approximately 25 to 50 mm. Below a microscope, the cells are dyed, fixed, and subsequently visually inspected [20]. Cytotechnologists, or Cytotec's for short, do the screening by looking for cancer indications in a cell sample under a light microscope. Through this process, they can identify evidence of invasive cancer and specific cancer precursors, enabling prompt and efficient treatment. It is reported when they discover something on a sample that is suggestive of malignancy. Usually, a woman with a rising premalignant lesion is given a pelvic exam and, if a lesion is found, surgery to correct it. If a low-grade lesion is found, it could be necessary to conduct a follow-up screening sooner than the usual 2-3 years [20]. The high rate of entirely bogus and factually inaccurate cervical smears, which causes an excess of medical testing or even a delay in the spotting of cervical cancer, is just a drawback of the current screening strategy [21-23]. Although it is predicted that receiving an HPV vaccine will lower recipients' chance of developing cervical cancer, current guidelines for screening tests need not take a woman's immunization status into account [17]. The low utilization of HPV vaccination in the United States, below 50% for completion of all three doses; the variable quality of documentation for a person's vaccination history (i.e., type of vaccine received, vaccination age, dosage timing); and the sparse data on the real-world effectiveness of vaccination in reducing infection and pre-cancer prevalence are some of the arguments against changing screening policy in HPV-vaccinated women. Figure 1 depicts the cervical cancer screening method.

Vaccination and prevention

Identifying hrHPV as the essential factor in carcinoma development has resulted in ground-breaking improvements in preventing cervical cancer, including preventive HPV vaccination. Given the fundamental evolutionary biology of HPV infection and cervical malignancy, the necessity of infection prevention in adolescent students who have been the least likely to be exposed, and the high levels of effectiveness and consequent expenditure of HPV vaccine [8,24]. Identifying hrHPV as the essential factor in the development of cervical cancer has resulted in ground-breaking improvements in preventing cervical cancer, including preventive HPV vaccination. Given the fundamental natural history of HPV infection and cervical tumorigenesis, the necessity of infection prevention in early adolescents who are the least likely to have been exposed, and the high levels of efficacy and consequent cost-effectiveness of the HPV vaccine [25]. HPV vaccines have been designed to target up to 9 HPV subtypes (quadrivalent vaccine, now supplanted by a nonavalent vaccine) [26]. The vaccinations, which are given to teenagers of both sexes, have been demonstrated to reduce the prevalence of HPV and CIN, with rates of up to 100% effectiveness against the vaccine-specific HPV strains and associated illnesses in women who have never been exposed [27]. In clinical studies, the vaccinations showed >90% effectiveness against pre-cancers and persistent HPV infections in HPV-negative people who finished the three-dose regimen [28]. Most industrialized nations have adopted national vaccination programs for adolescent females since immunizations are most effective when given to young people before HPV exposure. The 2vHPV vaccine was recently designed to represent the 4vHPV in the immunization program in Norway, where all three HPV immunizations are available [29].

Cervical cancer avoidance

There are two types of cervical cancer prevention: primary and secondary. Safer sexual behaviors, such as using condoms correctly and consistently to avoid cervix HPV infection, are essential to the primary prevention of the disease. The recently released HPV vaccinations Cervarix (GlaxoSmithKline, Brentford, UK) and Gardasil (Merck Sharp & Dohme Corp, New Jersey, US) may also help with the primary prevention of cervical cancer [30]. A customized mix of immunization, screening, and early detection according to the individual's unique circumstances will be the best effective prevention for every man or woman in this age range [31].

Educating people

Health-seeking behavior is impacted by awareness of the many cervical cancer indications, symptoms, and risk factors. Furthermore, the chance of contracting human papillomavirus (HPV) may be substantially decreased given their awareness of and favorable attitudes toward cervical cancer vaccination [1,5]. Numerous studies have revealed that women in various contexts in India have a low understanding of cervical cancer, prevention, and screening in addition to the absence of a screening and treatment effort for the disease [32]. According to surveys, even while some women were aware that cervical cancer might develop, only a small number of them were properly informed about the disease. Most women knew nothing about the sort of cancer, including not even its name, while the remainder had just heard the word of it [33]. One-fourth of high- and upper-middle-income nations (HIC/UMIC) had national HPV vaccination programs in place by the end of 2008, while there had been none in areas in low-income and lower-middle-income countries (LIC/LMIC). According to a new analysis, just 1.1% of girls between the ages of 10 and 20 in all 84 LIC/LMIC had received one or increased doses of the HPV vaccine by 2014. More than half (70 percent) of cervical cancer diagnoses have taken place in nations without a countrywide HPV vaccination program [25]. In a recent analysis that focused on the influence of day-to-day activities, variables on cancer fighters, the adverse effects of overweight and cigarettes on cancer recurrence, and survival rates in clinical trials in patients having breast, prostate, and colon malignancies were emphasized [34]. These changes are particularly significant since they help people adopt a better lifestyle, live longer, and avoid developing malignant conditions [35].

Barriers and affecting factors

**Figure 2:**
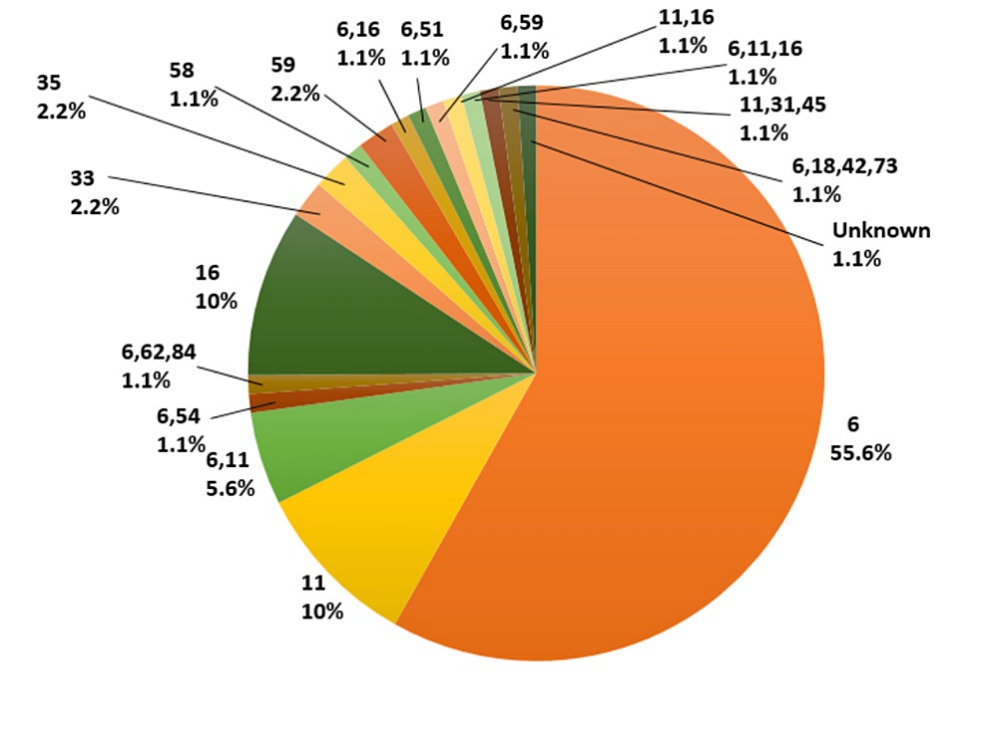
Frequency of different genotypes. This figure has been taken from source [16].

Programs for cervical screening in underdeveloped nations were not previously given attention. Prior to developing community-based screening programs, it is crucial to investigate variables and challenges related with cervical screening uptake [23]. The variables and constraints for cervical screening adoption across various nations have been identified through primary investigations. In order to enhance screening uptake, systematic studies of a variety of factors, including a special health promotion event, were also carried out. Self-collection of interventions and human papillomavirus (HPV) testing. A thorough analysis of the obstacles to cancer screening participation in industrialized nations, including the UK, Australia, Sweden, and Korea, was conducted. Reviews of the sub-Saharan African and Asian regions' challenges to cervical screening have been combined [36]. Low- and middle-income nations were not the primary focus of these systematic assessments. We undertook a thorough assessment of papers from low- and medium-income countries since the variables and barriers there are expected to differ greatly from those in rich nations [37]. Figure 2 depicts the frequency of different genotypes.

## Conclusions

The Pap smear test is a crucial and effective way to check for cervical cancer. Precancerous lesions are treated, and women are screened for the condition using Pap smears globally. In certain industrialized nations, Pap smear screening has shown impressive success in lowering cervical cancer mortality and incidence. Wherever screening quality and reliability are good, the cases of this type of cancer can be decreased by up to 90%. However, numerous women have never had a Pap smear in advanced countries, which accounts for 80% of all new cases. Screening programs must be appropriately implemented to lower the disease rate and severity of cancer in India. The study found that college students generally have a poor grasp of cervical cancer, HPV, and HPV vaccination. This comprehension is significantly connected to the student's age and biology-related educational background. There is an urgent need for educational intervention, both formal and informal, for both girls and boys with backgrounds in biology and non-biology, as well as for parents who wish to change their attitudes against HPV vaccination. To do this, print and audio-visual media may both be crucial. It is advised that the National Immunization Program include HPV vaccination so that it may be integrated with the screening (via pap/HPV DNA) and national cancer control programs, reducing the hurdles of vaccine cost and poor awareness for efficient prevention.

## References

[1] Dahiya N, Aggarwal K, Singh MC, Garg S, Kumar R (2019). Knowledge, attitude, and practice regarding the screening of cervical cancer among women in New Delhi, India. Ci Ji Yi Xue Za Zhi.

[2] Bhatla N, Aoki D, Sharma DN, Sankaranarayanan R (2018). Cancer of the cervix uteri. Int J Gynaecol Obstet.

[3] Sadia H, Shahwani IM, Bana KF (2022). Risk factors of cervical cancer and role of primary healthcare providers regarding PAP smears counseling: Case control study. Pak J Med Sci.

[4] Burd EM (2003). Human papillomavirus and cervical cancer. Clin Microbiol Rev.

[5] Nigussie T, Asefa A, Nigusse A, Admassu B (2020). Knowledge toward cervical cancer and its determinants among women aged 30-49 in Jimma Town, Southwest Ethiopia. Cancer Control.

[6] Franco EL (1995). Cancer causes revisited: human papillomavirus and cervical neoplasia. J Natl Cancer Inst.

[7] Dhanasekaran K, Verma C, Kumar V, Hariprasad R, Gupta R, Gupta S, Mehrotra R (2019). Cervical cancer screening services at tertiary healthcare facility: An alternative approach. Asian Pac J Cancer Prev.

[8] Chilaka VN, Navti OB, Al Beloushi M, Ahmed B, Konje JC (2021). Human papillomavirus (HPV) in pregnancy - An update. Eur J Obstet Gynecol Reprod Biol.

[9] Hong JN, Berggren EK, Campbell SL, Smith JS, Rahangdale L (2014). Abnormal cervical cancer screening in pregnancy and preterm delivery. Paediatr Perinat Epidemiol.

[10] Nygård M, Daltveit AK, Thoresen SO, Nygård JF (2007). Effect of an antepartum Pap smear on the coverage of a cervical cancer screening programme: a population-based prospective study. BMC Health Serv Res.

[11] Grellier N, Quéro L (2014). Cervical cancer: Particularities in HIV patients. Bull Cancer.

[12] Campaner AB, Fernandes GL (2021). Cervical cancer screening of adolescents and young women: further evidence shows a lack of clinical value. J Pediatr Adolesc Gynecol.

[13] William W, Ware A, Basaza-Ejiri AH, Obungoloch J (2019). A pap-smear analysis tool (PAT) for detection of cervical cancer from pap-smear images. Biomed Eng Online.

[14] Lynge E, Rygaard C, Baillet MV, Dugué PA, Sander BB, Bonde J, Rebolj M (2014). Cervical cancer screening at crossroads. APMIS.

[15] Bazaz M, Shahry P, Latifi SM, Araban M (2019). Cervical cancer literacy in women of reproductive age and its related factors. J Cancer Educ.

[16] Othman A, Goreal A, Pity I (2022). Molecular detection of human papilloma viruses in formalin fixed paraffin embedded sections from different anogenital lesions in Duhok-Iraq. Diagnostics (Basel).

[17] Kim JJ, Burger EA, Sy S, Campos NG (2017). Optimal cervical cancer screening in women vaccinated against human papillomavirus. J Natl Cancer Inst.

[18] Hu Z, Ma D (2018). The precision prevention and therapy of HPV-related cervical cancer: new concepts and clinical implications. Cancer Med.

[19] Nijhuis ER, Reesink-Peters N, Wisman GB (2006). An overview of innovative techniques to improve cervical cancer screening. Cell Oncol.

[20] Bengtsson E, Malm P (2014). Screening for cervical cancer using automated analysis of PAP-smears. Comput Math Methods Med.

[21] Basen-Engquist K, Paskett ED, Buzaglo J, Miller SM, Schover L, Wenzel LB, Bodurka DC (2003). Cervical cancer. Cancer.

[22] Boddu A, Bhatla N, Vashist S (2021). Cervical cancer screening in HIV-positive women in India: Why, when and how?. J Obstet Gynaecol India.

[23] Devarapalli P, Labani S, Nagarjuna N, Panchal P, Asthana S (2018). Barriers affecting uptake of cervical cancer screening in low and middle income countries: A systematic review. Indian J Cancer.

[24] Rosalik K, Tarney C, Han J (2021). Human papilloma virus vaccination. Viruses.

[25] Gallagher KE, LaMontagne DS, Watson-Jones D (2018). Status of HPV vaccine introduction and barriers to country uptake. Vaccine.

[26] Spayne J, Hesketh T (2021). Estimate of global human papillomavirus vaccination coverage: analysis of country-level indicators. BMJ Open.

[27] Sawaya GF, Huchko MJ (2017). Cervical cancer screening. Med Clin North Am.

[28] Kohler RE, Roncarati JS, Aguiar A, Chatterjee P, Gaeta J, Viswanath K, Henry C (2021). Trauma and cervical cancer screening among women experiencing homelessness: A call for trauma-informed care. Womens Health (Lond).

[29] Pedersen K, Burger EA, Nygård M, Kristiansen IS, Kim JJ (2018). Adapting cervical cancer screening for women vaccinated against human papillomavirus infections: The value of stratifying guidelines. Eur J Cancer.

[30] Shepherd JP, Frampton GK, Harris P (2011). Interventions for encouraging sexual behaviours intended to prevent cervical cancer. Cochrane Database Syst Rev.

[31] Wain G (2010). The human papillomavirus (HPV) vaccine, HPV related diseases and cervical cancer in the post-reproductive years. Maturitas.

[32] Reichheld A, Mukherjee PK, Rahman SM, David KV, Pricilla RA (2020). Prevalence of cervical cancer screening and awareness among women in an urban community in South India-A cross sectional study. Ann Glob Health.

[33] Nwabichie CC, Manaf RA, Ismail SB (2018). Factors affecting uptake of cervical cancer screening among African women in Klang Valley, Malaysia. Asian Pac J Cancer Prev.

[34] Schlumbrecht MP, Sun CC, Huang MS, Zandstra F, Bodurka DC (2014). Lifestyle modification in cervical cancer survivors: an ongoing need. Int J Gynecol Cancer.

[35] Datchoua Moukam AM, Embolo Owono MS, Kenfack B, Vassilakos P, Petignat P, Sormani J, Schmidt NC (2021). "Cervical cancer screening: awareness is not enough". Understanding barriers to screening among women in West Cameroon-a qualitative study using focus groups. Reprod Health.

[36] Dahlman D, Li X, Magnusson H, Sundquist J, Sundquist K (2021). Cervical cancer among Swedish women with drug use disorders: A nationwide epidemiological study. Gynecol Oncol.

[37] Ebu NI, Ogah JK (2018). Predictors of cervical cancer screening intention of HIV-positive women in the central region of Ghana. BMC Womens Health.

